# An artificial immune system with bootstrap sampling for the diagnosis of recurrent endometrial cancers

**DOI:** 10.1515/med-2021-0226

**Published:** 2021-01-29

**Authors:** Chih-Yen Chang, Yen-Chiao (Angel) Lu, Wen-Chien Ting, Tsu-Wang (David) Shen, Wen-Chen Peng

**Affiliations:** Department of Medical Education and Research, Jen-Ai Hospital, Taichung, Taiwan; Department of Elderly Care, Central Taiwan University of Science and Technology, Taichung, Taiwan; School of Nursing, College of Medicine, Chung-Shan Medical University, Taichung, Taiwan; Division of Colorectal Surgery, Department of Surgery, Chung Shan Medical University Hospital, Taichung, Taiwan; Department of Automatic Control Engineering, Feng Chia University, No. 100, Wenhwa Road, Seatwen, Taichung, 40724, Taiwan; Master’s Program in Biomedical Informatics and Biomedical Engineering, Feng Chia University, No. 100, Wenhwa Road, Seatwen, Taichung, 40724, Taiwan; Department of Long-Term Care, Jen-Ai hospital, Taichung, Taiwan

**Keywords:** artificial immune systems, bootstrap sampling, biomimetic intelligence, recurrent endometrial cancers

## Abstract

Endometrial cancer is one of the most common gynecological malignancies in developed countries. The prevention of the recurrence of endometrial cancer has always been a clinical challenge. Endometrial cancer is asymptomatic in the early stage, and there remains a lack of time-series correlation patterns of clinical pathway transfer, recurrence, and treatment. In this study, the artificial immune system (AIS) combined with bootstrap sampling was compared with other machine learning techniques, which included both supervised and unsupervised learning categories. The back propagation neural network, support vector machine (SVM) with a radial basis function kernel, fuzzy c-means, and ant k-means were compared with the proposed method to verify the sensitivity and specificity of the datasets, and the important factors of recurrent endometrial cancer were predicted. In the unsupervised learning algorithms, the AIS algorithm had the highest accuracy (83.35%), sensitivity (77.35%), and specificity (92.31%); in supervised learning algorithms, the SVM algorithm had the highest accuracy (97.51%), sensitivity (95.02%), and specificity (99.29%). The results of our study showed that histology and chemotherapy are important factors affecting the prediction of recurrence. Finally, behavior code and radiotherapy for recurrent endometrial cancer are important factors for future adjuvant treatment.

## 1 Introduction

Endometrial cancer is the sixth most common cancer among women worldwide [1]. According to a report of the International Agency for Research on Cancer, there were over 3,80,000 women living with endometrial cancer worldwide in 2018 [2].

In Taiwan, endometrial cancer is the most common neoplasm of the female genital tract [3]. According to a report by the Taiwan Cancer Registry, the annual incidence rate of endometrial cancer was 2,462 cases per 1,00,000 women in 2016 compared to 399 per 1,00,000 in 1996. It is estimated that there will be more than 3,000 new cases in 2020. Factors that influence endometrial cancer survival are of increasing importance as lifestyle-related mortality risk factors for this population may differ from those of the general population. The 5-year survival for endometrial cancer depends on the cancer stage at diagnosis. In early-stage disease, surgery alone or in combination with local therapy is generally curative [4,5]. If primary treatment fails, the opportunity for a secondary cure is slim. The treatment of endometrial cancer requires a complex therapeutic approach, consisting of surgery, radiotherapy, chemotherapy, and/or hormonal therapy. All patients are usually classified further based on the extent or stage of the disease so that therapies may be tailored to the particular disease stage. The choice of therapy depends on the extent of residual disease after initial surgery, the site and nature of recurrence, the prior therapy used, and the intent of treatment, be it curative or palliative. The risk factors for recurrent endometrial cancer include obesity, diabetes, late menopause, unopposed estrogen therapy, and nulliparity [6,7]. Inherited factors have also been suggested as important risk factors for recurrence [8]. The goal of this study was to use a biomimetic algorithm to select important factors for the diagnosis of recurrent endometrial cancers, investigate the risks associated with endometrial cancer, and provide appropriate primary treatment to enable the management of recurrent endometrial cancer.

## 2 Materials and methods

The datasets were provided by the cancer registry of the Cancer Center of Chung Shan Medical University Hospital under regulation. This study used a total of 599 valid records obtained from the endometrial cancer dataset provided by the Cancer Center of Chung Shan Medical University Hospital. The research process of this study was as follows: First, an endometrial cancer dataset was obtained from the cancer registration centers. Second, clinical experts provided important recurrent candidate variables and reviewed previous studies regarding this topic. Third, the dataset was cleaned and recoded. The exclusion criteria were as follows: patients with malignant disease of reproductive organs other than endometrial cancer, those previously treated for cancer, and those diagnosed within the previous 5 years.

There were a total of 20 predictive variables in the dataset as follows: (1) age (52.73 ± 11.82 years), (2) histology, (3) behavior code, (4) grade, (5) tumor size, (6) stage, (7) surgery, (8) radiotherapy, (9) surgical margin, (10) chemotherapy, (11) sequence of locoregional therapy and systemic therapy, (12) the highest radiation dose clinical target volume dose (dose to CTV_H: cGy), (13) clinical target volume treatment times of maximum radiation dose (number of fractions to CTV_H), (14) lower radiation dose clinical target volume dose (dose to CTV_L: cGy), (15) clinical target volume treatment times of lower radiation dose (number of fractions to CTV_L), (16) sequence of radiotherapy and surgery, (17) body mass index (BMI) (higher or lower than 24), (18) smoking, (19) betel nut chewing, and (20) drinking behavior. One target variable (type of recurrence) was used for the prediction of recurrence for a total of 599 sets of data. In this study, the artificial immune system (AIS) was compared with other machine learning techniques, including both supervised and unsupervised learning categories. The back propagation neural network (BPNN) and support vector machine (SVM) with a radial basis function kernel are types of supervised learning; the fuzzy c-means (FCM), ant k-means (AK), and AIS algorithms are types of unsupervised learning. The details of each are described in this section.

### 2.1 Back propagation artificial neural network

The BPNN [9] is an artificial neural network that combines the feedforward full connection of neurons with feedback loops. The BPNN consists of three layers, namely, the input, hidden, and output layers. The feedback loops, the so-called backward propagation, adjust the weights to achieve the minimum optimal solution of error between the output and desired signal in the mean square sense. Finally, the weights of each unit update according to the gradient. The BPNN structure for classification was *N*-15-2, meaning that the number of features was *N* with 15 neurons in the hidden layer and two neurons in the output layer, that is, two-digit outputs (01 and 10) represent diagnosis results to reduce the decision noise. A sigmoid function was used as an activation function.

### 2.2 Support vector machine

The SVM [10] is a supervised learning method that analyzes data and recognizes patterns. The SVM algorithm is based on the structural risk minimization principle of statistical learning theory. The algorithm seeks a separating hyperplane to classify groups with corresponding binary labels with a maximal margin and minimal error in the support vector sense. The optimal separating hyperplane maximizes the gaps among the support vectors. The positive and negative samples train a classifier to map the input samples to another space using a kernel function that can map data into another infinite-dimensional space. The decision function is as follows:

(1) f(x)=\mathrm{sgn}\left(\mathop{\sum }\limits_{i=1}^{m}{\alpha }_{i}{d}_{i}\hspace{.5em}K(x,{x}_{i})+b\right)

where *K*(*x*, *x*_*i*_) is the radio basis function kernel function and the dual problem provides the solution of the Lagrange multiplier problem at a saddle point parameter. The regularization parameter controls the trade-off between the margin and classification error. The SVM engine was implemented using the LIBSVM package [11]. K-fold data validation was applied to evaluate the SVM system performance.

### 2.3 Fuzzy c-means

FCM is an unsupervised learning model inherent from the k-means algorithm [9,13] added to fuzzy logic decision sets. However, instead of binary indicators, FCM uses probability indicators to determine the degrees of membership. This method identifies the minimum distance between the input vector and specific classes. The main procedures are as follows: (1) initialize the indicators to make the sum of indicators equal to one, (2) calculate the codebooks using indicators and input vectors, (3) re-compute the new indicators by applying new codebooks, (4) calculate the distance of the FCM of each group, and (5) repeat the previous steps until all codebooks converge.

### 2.4 Ant colony optimization (ACO) algorithm

ACO is an unsupervised nature computing approach that is a recently proposed metaheuristic for solving hard combinatorial optimization problems [12]. In particular, the AK clustering method is one branch of ACO approaches proposed by Kuo et al. [13]. This robust clustering method provides a self-organized structure that simulates the interaction of an ant society to solve optimization problems. Instead of computing the distances toward the center codebooks, the method applies a certain probability, called pheromone, which is a biochemical material for tracking other ants. Repeated feature information provides a higher pheromone concentration to guide ants toward their targets. On the contrary, pheromones naturally evaporate over time, so that longer travel paths can cause low pheromone concentrations. Therefore, the optimal path with a higher pheromone is guaranteed. The AK algorithm assigns each data point to a specific cluster (class) based on the probability provided by the pheromone from each ant. After iterations, the optimal solution converges based on the in-grouped distances and pheromone concentrations.

### 2.5 AIS algorithm

The AIS algorithm [14] is based on an unsupervised artificial immune classifier with hormone concentration. The algorithm is a mathematical model to mimic clonal selection theory for selecting the best affinity antibodies to handle specific antigens as foreign substances, where antigens or foreign substances are the input data for clustering. If the affinity between antibodies and antigens is high, antibodies result in the production of mutated clones against antigens. B cells save memory on various antibodies for immediate response upon future invasion by the same antigens.

**Figure 1:**
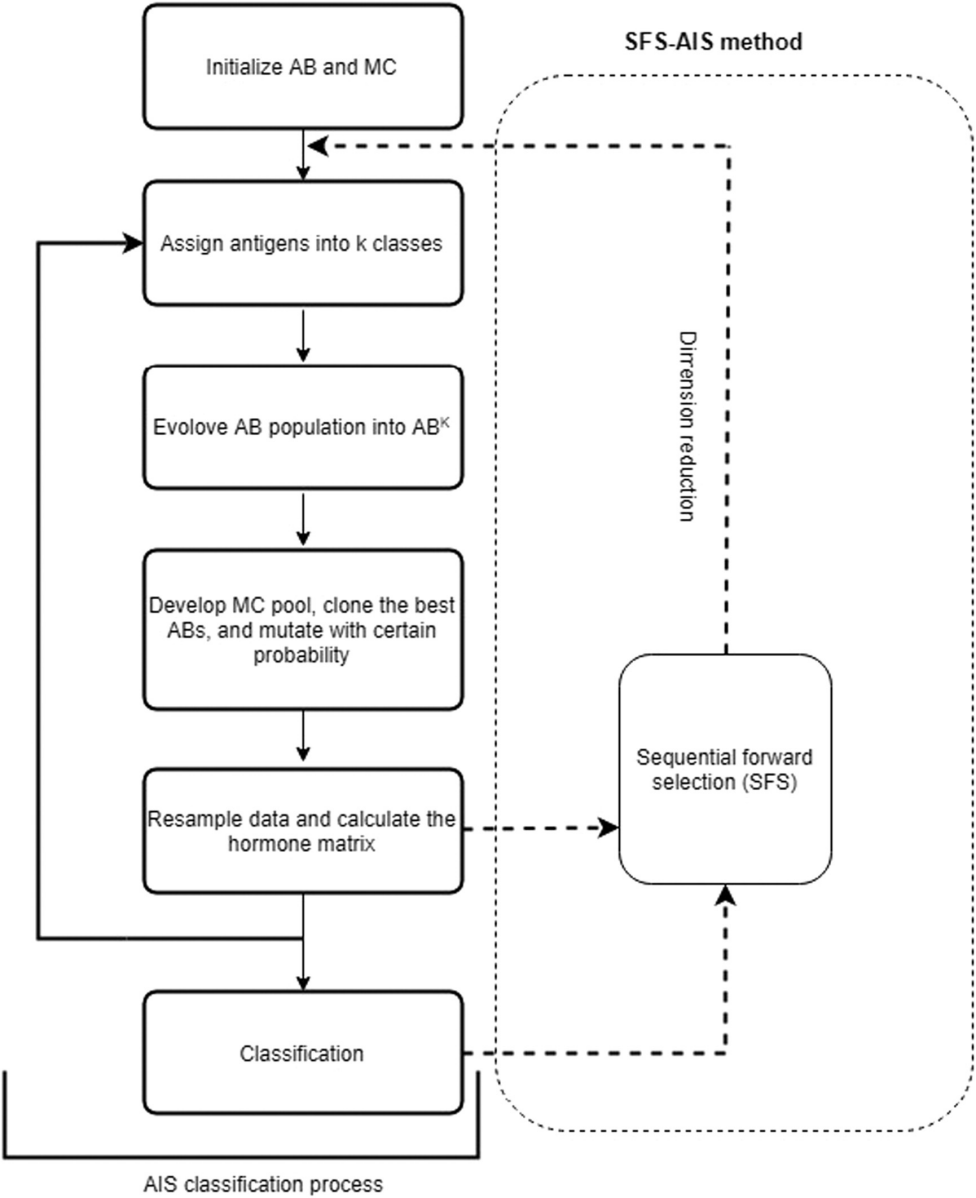
AIS classification process and SFS-AIS process.

Based on these physiological facts, the algorithm first applies k-means clustering to give initial center points of hormone and initial B-cell population. Then, the affinity is calculated within each class group antigen and the *n* highest affinity antigens (AG) to be antibodies (AB). The radius of influence is set to 0.1 in the system. The hormone matrix (HM) covers the entire antigen area, which provides the probability sense of hormone concentration. The selected *n* best ABs are used to generate a clonal set. In the clonal set, if the AB affinity is higher, ABs will clone more. The clonal rate is used to determine how many clones are produced by ABs and memory B-cells, and the round function is an argument toward the closest integer. The clonal rate in this system was 10. If the affinity was higher, the mutation (MU) rate decreased. AB and MU sets were compared to update the AB list. If the generated MU had a higher affinity in relation to AG than the previous AB, then MU replaced the previous AB to update the old AB list. Finally, we updated the MC population and hormone concentration matrix (HM) to classify data. After the AIS system convergence, the system used the MC set and HM to assign AG as a certain class. When the two decisions were matched, the classification was determined. If the decisions were conflicting, an AG in the burring area was determined by the closest MC. The burring area means that the difference in hormone concentration was small (within a certain radius *r*) by observing probabilities in the HM. Otherwise, the strongest hormone concentration is the deciding factor. Figure 1 shows the entire AIS process.

### 2.6 Bootstrap sampling method

The clinical dataset used was an imbalanced medical dataset, meaning that the number of one class was much greater than that of the other classes. To provide good classification performance in the class with fewer samples, a bootstrapping statistic technique was used to provide balanced datasets. This is a resampling method that generates a number of resamples with replacement for constructing an equal size to the observed dataset [15] in the data distribution sense. This technique measures original data properties, including variance bias, variance, confidence intervals, and prediction error, to estimate replacement samples when sampling from an approximating distribution.

### 2.7 The proposed feature selection method with comparison

Not all features have the same importance and may contain redundant or unrelated information and noise. Therefore, the goal of feature selection is to select the best one consisting of the original features so that the recognition rate can reach the global maximum. Good features with a better discriminating ability not only simplify the calculation of the classifier but also help understand the causal relationship of this classification problem. In addition, they speed up the training process and improve classification accuracy.

Our proposed method combines the AIS algorithm and the approximate optimal method, which is a sequential forward selection (SFS) [16], called SFS-AIS. The ABs, MCs, and HM of the AIS algorithm were mapped to the entire dataset, and SFS reduced the feature dimension. Therefore, the proposed method speeds up the training process and improves classification accuracy simultaneously. In Figure 1, the SFS-AIS steps include (1) computing AG, MC, and HM of AIS for classification; (2) leave-one-out to select the feature with the lowest recognition rate and eliminate the selected feature to improve the recognition rate; and (3) repetition of step one sequentially until 11 features were selected. Then, the proposed SFS-AIS method was compared to other feature selection methods, including the relief and information gain algorithm.

The relief algorithm is a filter method approach that provides ranking scores for feature selection. Its score is notably sensitive to feature neighborhoods and depends on the differences between the nearest neighbor vectors. The feature score decreases when a feature value difference is observed in a neighboring vector with the same class, called a hit. Alternatively, the feature score increases when a feature value difference is observed in a neighboring vector with different class values, the so-called miss. The advantages of this method are independent of heuristics, low-order polynomial time, noise tolerance, and robustness to feature interactions; however, it does not discriminate between redundant features, and low numbers of training instances fool the algorithm [17]. Finally, these scores may be applied as feature weights to inhibit bad features.

The information gain method ranks features based on entropy according to the information theory and is widely used in decision trees, such as ID3, C4.5, and C5.0. Information gain determines the most relevant attributes, and the highest information gains are the criteria of good features.

In this study, of the 20 features, 9 were dropped and the remaining 11 were selected as input features according to the above feature selection methods for comparison.

## 3 Results

**Figure 2:**
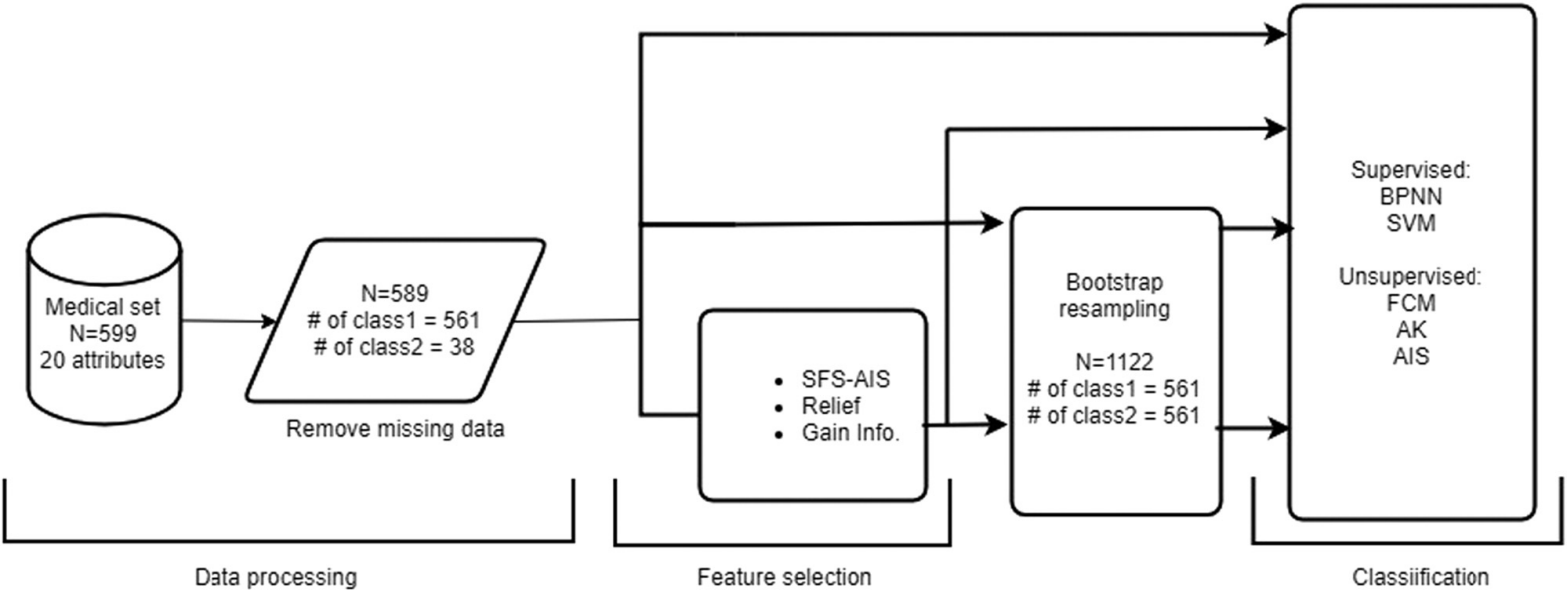
System analysis framework.

In this study, we used BPNN, SVM, FCM, AK, and AIS to verify the sensitivity and specificity of datasets provided by the Cancer Center of Chung Shan Medical University Hospital, and the important factors of recurrence were predicted. The system framework is shown in Figure 2. In the data-processing stage, the missing data were first removed from the dataset. Then, our proposed SFS-AIS, relief, and gain information algorithms were used as feature selection approaches to determine the best feature combination for every target variable. Different approaches have different strengths, and Table 1 lists the best combinations of the three different algorithms. We found that histology and chemotherapy were selected as being most important by all methods, which implies that these features are essential.

**Table 1:** Best feature combinations of three feature selection methods

Methods	Top 11 features
SFS-AIS	1, **2**, 4, 5, 6, **10**, 11, 12, 14, 18, 19
Gain info.	**2**, 3, 7, 8, **10**, 11, 12, 13, 14, 15, 20
Relief algorithm	1, **2**, 4, 5, 6, 8, 9, **10**, 13, 15, 17

The numbers represent features, including (1) age, (2) histology, (3) behavior code, (4) grade, (5) tumor size, (6) stage, (7) surgery, (8) radiotherapy, (9) surgical margin, (10) chemotherapy, (11) sequence of locoregional therapy and systemic therapy, (12) the highest radiation dose clinical target volume dose (dose to CTV_H: cGy), (13) clinical target volume treatment times of maximum radiation dose (number of fractions to CTV_H), (14) lower radiation dose clinical target volume dose (dose to CTV_L: cGy), (15) clinical target volume treatment times of lower radiation dose (number of fractions to CTV_L), (16) sequence of radiotherapy and surgery, (17) BMI, (18) smoking, (19) betel nut chewing, and (20) drinking behavior and one target variable (type of recurrence). The bold fonts present the common features of all methods.

After feature selection, bootstrap sampling was used to generate more data to balance both classes to obtain better system performance. The cancer group (*N* = 38) was resampled to match the control group with *N* = 561. Before bootstrap sampling, the overall sensitivity was quite low in Table 2 for all classification algorithms owing to data imbalance. That is, because of overfitting on one large number class, the small group could not be correctly identified and the accuracy rate had no significant meaning. According to the results in Table 2, the bootstrap sampling method essentially improved the system performance when accuracy, sensitivity, and specificity were considered. For the same 20 features, all positive predictive values in five classifiers increased significantly from 0, 13.89, 7.02, 24.32, and 29.73% to 94.27, 99.62, 83.13, 84.34, and 82.03%, respectively.

**Table 2:** Performances of all combinations

No bootstrap	BPNN	SVM	FCM	AK	AIS
Number of features	20	20	20	20	20
Accuracy (%)	93.59	76.51	69.23	71.07	70.07
Sensitivity (%)	aNaN	55.56	32.43	5.84	6.71
Specificity (%)	93.59	77.86	71.66	93.69	94.01
PPV (%)	0	13.89	7.02	24.32	29.73
NPV (%)	100	96.94	94.15	74.15	72.73

With bootstrap	BPNN	SVM	FCM	AK	AIS
Number of features	20	20	20	20	20
Accuracy (%)	81.87	96.79	74.18	72.31	74.80
Sensitivity (%)	75.54	93.95	60.61	68.01	71.70
Specificity (%)	92.39	99.64	87.72	79.34	78.96
PPV (%)	94.27	99.62	83.13	84.34	82.03
NPV (%)	69.47	94.26	60.61	60.25	67.56

No bootstrap	BPNN	SVM	FCM	AK	AIS
Number of features (SFS)	11	11	11	11	11
Accuracy (%)	93.59	68.64	65.22	58.36	66.22
Sensitivity (%)	aNaN	66.67	27.03	7.26	9.76
Specificity (%)	93.59	68.57	67.74	94.57	95.67
PPV (%)	0	12.00	5.24	48.65	54.05
NPV (%)	100	96.97	67.74	59.00	67.02

With bootstrap	BPNN	SVM	FCM	AK	SFS-AIS
Number of features (SFS)	11	11	11	11	11
Accuracy (%)	91.60	97.51	71.95	82.55	83.35
Sensitivity (%)	94.31	95.02	56.86	75.00	77.53
Specificity (%)	89.21	99.29	87.01	96.68	92.31
PPV (%)	88.55	99.26	81.38	97.69	93.95
NPV (%)	94.66	95.21	66.89	67.38	72.73

a NaN means Not a Number, in which true positive and false negative are equal to zero.

**Figure 3:**
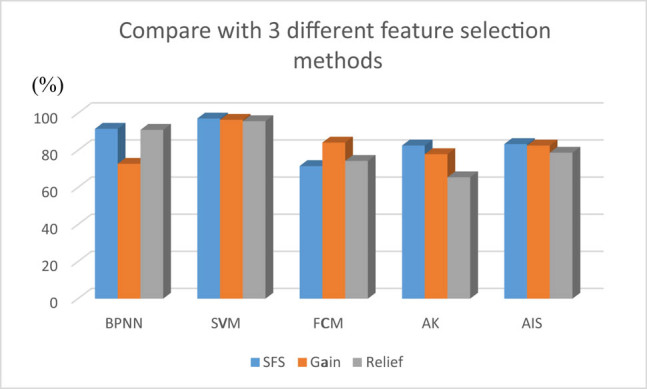
Comparison between proposed method (blue color bar), relief algorithm (orange color bar), and gain information algorithm (gray color bar).

Moreover, after applying the feature reduction methods to reduce the number of features from 20 to 11, the algorithm performance was further improved. The comparison results of the three feature selection methods are shown in Figure 3. After feature reduction, it was found that feature selection methods provided higher classification accuracy than all-feature classification accuracy. Our proposed SFS-AIS method generates the best feature combination to provide the best overall performance among BPNN, SVM, AK, and AIS classification methods of both supervised and unsupervised learning, except FCM method.

In the unsupervised learning algorithms, the AIS algorithm has the highest accuracy (83.35%), sensitivity (77.35%), and specificity (92.31%); in the supervised learning algorithms, the SVM algorithm has the highest accuracy (97.51%), sensitivity (95.02%), and specificity (99.29%). However, the AIS algorithm had no pre-training requirements and could adapt the unknown models without a training process. Unlike SVM, AIS can handle situations with an unknown number of classes. Therefore, the AIS algorithm could become a general proposal for artificial intelligence for future medical diagnosis.

## 4 Discussions

Several studies have used variable observations, such as outpatient prescriptions and treatment regimens from the National Health database, for data analysis. However, to increase the cured and survival rates, it is crucial to identify factors predicting recurrence in actual diagnosis and treatment records. To obtain better important factors for recurrence, this study used multiple feature selection methods to identify the risk factors for recurrence. The SFS-AIS method provided the best feature combinations among the three feature selection methods.

The results of this study showed that histology [18] and chemotherapy [19] were important factors affecting the prediction of recurrence. In addition, early diagnosis of recurrence should not be neglected in the treatment of radiotherapy [20] and surgery [21], and long-term follow-up should be considered [22]. In particular, older patients with endometrial carcinoma are more likely to fare worse than younger patients, independent of other poor prognostic factors [23]. Similarly, the behavior code [24] appeared to be correlated with recurrence. In addition, patients with a BMI < 24 had a lower probability of developing recurrence than those with a BMI ≥ 24 [25].

**Figure 4:**
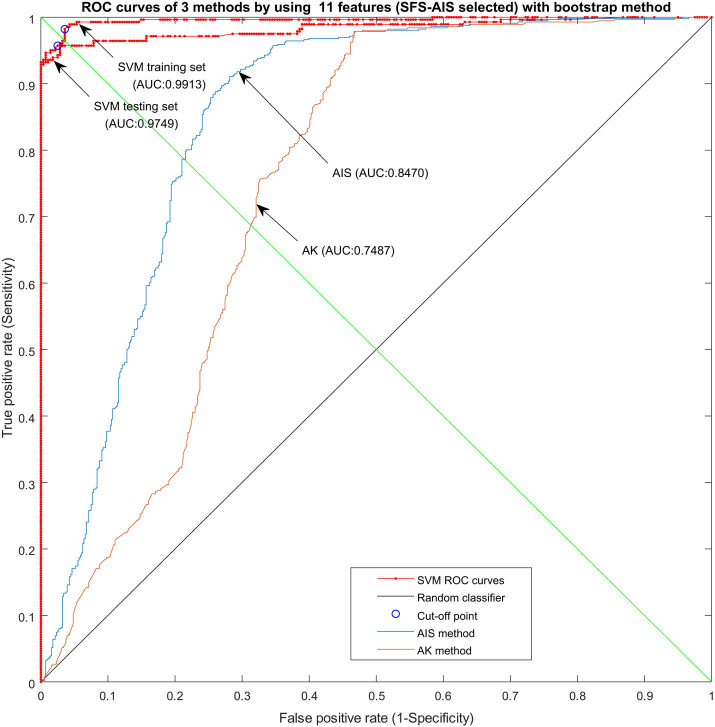
ROC curves of three methods (SVM, AIS, and AK) using 11 features (SFS-AIS selected) with bootstrap method. SVM provides the best performance among supervised learning methods and AIS provides the best performance among unsupervised learning method.

Further comparison of the predictive accuracy of BPNN, SVM, FCM, AK, and AIS for endometrial cancer was carried out. As shown in Figure 4, SVM and AIS classification methods had the best performance in the supervised and unsupervised categories, respectively. The results of this retrospective study proved that for recurrence detection in patients with endometrial cancer, stratification by behavior code and radiotherapy could be used by clinicians to recommend adjuvant treatment. In the future, longitudinal studies may provide a better explanation of the long-term effects of treatments.

## 5 Conclusions

In Taiwan, endometrial cancer is the second most commonly diagnosed gynecologic malignancy, following cervical cancer of the female genital tract. According to the latest cancer statistical report, more than 3,200 new cases of endometrial cancer are expected to be diagnosed in 2020. In this study, the SFS-AIS feature selection method combined with bootstrap sampling indicated that the unsupervised biomimetic AI system can efficiently refine the 20 features down to 11 well-performing features to improve the multiple classification methods. Overall, this study showed that combination therapy with age, histology, behavior code, and radiotherapy proved to be the optimal prediction parameters for patients with recurrent endometrial cancer. For a better understanding of the disease, considering the existence of selection bias, recurrence can be detected early and appropriate primary treatment can be commenced accordingly, to enable the management of recurrent endometrial cancer.
